# A Gamified, Social Media–Inspired, Web-Based Personalized Normative Feedback Alcohol Intervention for Lesbian, Bisexual, and Queer-Identified Women: Protocol for a Hybrid Trial

**DOI:** 10.2196/24647

**Published:** 2021-04-16

**Authors:** Sarah C Boyle, Joseph W LaBrie

**Affiliations:** 1 HeadsUp Labs Department of Psychology Loyola Marymount University Los Angeles, CA United States

**Keywords:** sexual minority women, alcohol, intervention, social norms, gamification, protocol, mobile phone

## Abstract

**Background:**

Sexual minority women are more likely to drink alcohol, engage in heavy drinking, and experience alcohol-related problems than heterosexual women. However, culturally tailored interventions for this population have been slow to emerge.

**Objective:**

This type 1 effectiveness-implementation trial examines the feasibility and efficacy of a gamified, culturally tailored, personalized normative feedback (PNF) alcohol intervention for sexual minority women who psychologically identify as lesbian, bisexual, or queer (LBQ).

**Methods:**

The core components of a PNF intervention were delivered within LezParlay, a fun, social media–inspired, digital competition designed to challenge negative stereotypes about LBQ women and increase visibility. The competition was advertised on the web through social media platforms and collaboration with LBQ community organizations. After 2 rounds of play by a large cohort of LBQ women, a subsample of 500 drinkers already taking part in the competition were invited to participate in the evaluation study. Study participants were randomized to receive 1 of 3 unique sequences of PNF (ie, alcohol and stigma coping, alcohol and control, or control topics only) over 2 intervention rounds. Randomization was fully automated by the web app, and both researchers and participants were blinded.

**Results:**

Analyses will evaluate whether PNF on alcohol use reduces participants’ drinking and negative consequences at 2 and 4 months postintervention; examine whether providing PNF on stigma-coping behaviors, in addition to alcohol use, further reduces alcohol use and consequences beyond PNF on alcohol alone; identify mediators and moderators of intervention efficacy; and examine broader LezParlay app engagement, acceptability, and perceived benefits.

**Conclusions:**

This *incognito* intervention approach is uniquely oriented toward engaging and preventing alcohol-related risks among community populations of LBQ women who may view their heavy drinking as normative and not in need of change because of the visibility of alcohol use in sexual minority community spaces. Thus, this intervention strategy diverges from, and is intended to complement, more intensive programs being developed to meet the needs of LBQ women already motivated to reduce their consumption.

**Trial Registration:**

ClinicalTrials.gov NCT03884478; https://clinicaltrials.gov/ct2/show/NCT03884478

**International Registered Report Identifier (IRRID):**

DERR1-10.2196/24647

## Introduction

### Background

The Institute of Medicine first identified sexual minority women (SMW) as a medically underserved population that disproportionately engage in hazardous drinking and carry the burden of alcohol dependence two decades ago [1]. At present, sexual orientation–based alcohol-use disparities remain with national survey data, revealing lesbian and bisexual women to be among the heaviest drinking female populations [2-4]. For instance, compared with heterosexual women of the same age, lesbian and bisexual women are more than twice as likely to engage in heavy drinking [4,5], 4-7 times more likely to meet the Diagnostic and Statistical Manual of Mental Disorders criteria for alcohol dependence [5,6], and 9-11 times more likely to report that their drinking has led to serious interpersonal consequences [5,6]. In addition, relative to their sexual majority female peers, SMW are more likely to continue patterns of heavy drinking as they age [7,8], increasing their risk for several cancers [8,9] and cardiovascular diseases [10,11]. Despite these striking disparities and consequences, targeted alcohol intervention and prevention efforts for SMW have been slow to emerge [12,13].

Recent research suggests that personalized normative feedback (PNF) may represent a welcomed low-risk, high-reward strategy for reducing alcohol use among SMW, particularly those who psychologically identify as lesbian, bisexual, or queer (LBQ) [14-16]. Founded in the social norms approach [17,18], modern PNF interventions first prompt nontreatment-seeking members of a target group or community to answer a series of web-based survey questions about their perceptions of peers’ alcohol use and their own drinking. Group members then receive individualized digital reports that use bar charts to highlight discrepancies among their perceptions of peers’ drinking, peers’ actual drinking, and their own drinking [19]. To increase the appeal and cost-effectiveness of PNF for this population, we introduced a culturally tailored, social media–inspired, gamified intervention format designed to simultaneously engage heavy drinking LBQ women just as well as their lighter-drinking and alcohol-abstaining peers and remedy the oft-cited attentional and motivational limitations associated with traditional web-based PNF intervention formats.

### Why a Brief, Social Norms–Based Alcohol Intervention for SMW?

Although social norms are among the most predictive and commonly targeted antecedents to alcohol use in other heavy drinking populations [20-23] and a growing body of the literature identifies sexual minority–specific peer substance use norms as appropriate targets for intervention and prevention efforts [14,15,24-27], the dominant perspective for understanding heavy drinking among SMW is not that of social norms but rather of sexual minority stress [28-30]. This model emphasizes the role of stigma in SMW’s increased alcohol use, explaining that the increased drinking and dependence among SMW may derive from separate and combined effects of distal stressors, including experiences of prejudice, rejection, harassment, discrimination, and violence originating from a heterosexual society [28,30,31], as well as proximal stressors rooted within the individual, including internalization of stigma, concealment, and inadequate or problematic coping. Growing support for the link between sexual minority stress exposure and coping-motivated drinking [32-38] has encouraged the development of intensive, sexual identity–affirming programs that seek to reduce alcohol use and improve mental health by increasing individuals’ understanding of stigma-related processes and bolstering their adaptive coping skills and resources [39,40]. Despite the promise of these interventions for SMW seeking treatment for their problematic drinking [41,42], these strategies are unlikely to engage the larger population of SMW who do not view their drinking as problematic or lack motivation to reduce their consumption.

In contrast, web-based PNF interventions have the potential to cost-effectively reach and motivate reductions in drinking among SMW not seeking treatment and those who do not yet view their drinking as excessive or a risk to their overall health. Similar to the heavy drinking populations of college students [20,43], military personnel [21,22], and working adults [44] commonly targeted by web-based PNF alcohol interventions, bisexual- and lesbian-identified SMW have been found to overestimate descriptive peer drinking norms [13,14]. Over time, these perceptions of norms have been found to relate to drinking behavior among lesbian and bisexual SMW in the standard reciprocal feed-forward fashion observed in other groups for whom PNF has been effective [15]. Building on these findings, the primary aim of this trial is to examine the extent to which PNF designed to correct sexual identity and age-specific drinking norms is efficacious in augmenting these normative perceptions, thereby reducing LBQ women’s alcohol use and negative consequences. Recent research also suggests that in addition to general peer drinking norms (ie, frequency or quantity of consumption), this population also overestimates peer norms specific to coping-motivated drinking following collectively experienced sexual minority stressors such as the Pulse Nightclub shooting [45] and the 2016 US presidential election [46], with these misperceptions also contributing to their current and future drinking beyond the self-reported stress impact of these events. As such, the secondary aim of this trial is to evaluate whether delivering PNF on stigma-coping behaviors, in addition to alcohol use, further reduces alcohol use and consequences beyond PNF on alcohol use alone.

### Testing the Boundaries of PNF Efficacy Among SMW

Few longitudinal studies have simultaneously examined the perceptions of sexual identity–specific substance use norms and concurrently experienced sexual minority stressors as predictors of substance use [47], and no published studies investigating alcohol use among SMW have considered how these predictors may influence or interact with one another over time. Rather, sexual minority stress and stigma have long been positioned in the literature as primary targets for substance use interventions, and the implicit perspective among many lesbian, gay, and bisexual health researchers seems to be that social disadvantage and stigma-related processes may render evidence-based intervention strategies that are effective in other populations ineffective among sexual minorities. Indeed, as the sexual minority stress model [30] positions sexual minority communities as stress-buffering resources and does not consider how perceptions of substance use norms may be artificially inflated by these social environments, it is certainly possible that sexual minority stress and stigma-related processes may render a PNF alcohol intervention ineffective.

However, 2 longstanding social psychological theories support the prediction that correcting misperceived sexual identity–specific drinking norms may be maximally effective among SMW who experience severe interpersonal sexual minority stressors such as violence and harassment because of the sexual minority status. Specifically, self-categorization theory [48-50] contends that when an intergroup threat is experienced in a self-relevant domain, as would be the case when an LBQ woman experiences prejudice, harassment, or violence because of her sexual minority status, she will be particularly likely to turn to perceived in-group norms to guide her behavior. Similarly, terror management theory [51-53] posits that embracing the cultural standards and norms of a self-relevant group can protect against the deeply rooted fears of mortality likely to arise from such threatening intergroup experiences. Thus, following from both theories, to the extent that an LBQ woman experiences prejudice or victimization because of her sexual identity and has inflated perceptions of how much same-identity peers drink, she may be especially likely to increase her drinking as a means of conforming to this inflated normative standard. However, if PNF were to correct her misperceptions of these norms, she should also be especially motivated to align her drinking to the risk-reducing *true norm*. This study directly examines whether interpersonal stigma exposure moderates the efficacy of PNF designed to correct misperceived norms for LBQ peers’ drinking and coping behaviors.

Additional potential moderators of PNF intervention efficacy examined in this trial include preintervention alcohol consumption and negative consequences, sexual identity, age, race or ethnicity, and relationship status. Relative to lighter drinkers, heavier drinkers naturally have greater room for behavior change following PNF’s correction of drinking norms [19,22]. Thus, heavier drinking SMW are expected to exhibit larger reductions in their drinking post-PNF relative to their lighter-drinking peers. Although sexual identity, race or ethnicity, age, and relationship status are factors associated with variability in alcohol consumption among SMW [54], to date, no published studies have compared the relative sizes of discrepancies between the perceived and actual norms or the strengths of relationships between the perceived norms and alcohol consumption among subgroups of SMW who differ in these demographic characteristics. As such, this trial also evaluates whether these demographic characteristics moderate PNF intervention efficacy among LBQ women.

### Addressing PNF Intervention Limitations and Engaging a Hard-to-Reach Population

An extensive study of web-based PNF interventions among college students suggests that these interventions lead to reliable but relatively short-term and modest reductions in drinking [20]. Researchers have identified several issues that, if remedied, could considerably increase the impact of this strategy. In particular, doubts about the credibility of actual drinking norms derived from previously collected data sources [55,56], defensive reactions among heavy drinkers [57,58], general inattention to feedback [59,60], and low motivation among participants [61] have been proposed as barriers to greater public health impact. The real-world suitability of this approach has also drawn criticism [62], as researchers have struggled to implement web-based PNF interventions as well as engage and retain heavy drinkers outside of study settings where participation is mandatory or participants are offered compensation at the point of recruitment [61,63]. Beyond these issues, sexual minority communities present unique challenges for intervention dissemination. Unlike universities and military bases where mandatory PNF interventions can easily target new cohorts of students and recruits, there is no single institution from which SMW can be easily recruited. In contrast, SMW must hear about community programs and judge whether the programs are credible and worthwhile.

One tool commonly used to increase the credibility and appeal of health promotion programs for minority populations is cultural tailoring, which refers to the development of interventions, messages, and materials to conform with specific cultural characteristics of the target group [64]. Recommended cultural tailoring practices for SMW include the development of programs and materials that reflect the social identities, values, and lived experiences of LBQ women as well as the involvement of LBQ community members and trusted community organizations in program promotion and delivery [12,65,66]. Following these recommendations and seeking to bolster intervention relevance, engagement, and motivation, PNF designed to correct drinking and stigma-coping norms were delivered within a larger digital competition called LezParlay. This competition was strategically crafted to reflect deep-structure cultural themes, including community members’ awareness of negative LBQ stereotypes [67-69], desire for increased identity visibility [70-72], and enjoyment of intracommunity competition and sport [73-75]. Consistent with the recommendations for surface-structure intervention tailoring [12,65,76], the LezParlay competition was also developed by an LBQ woman in the target age range and jointly promoted on the web by 4 collaborating community organizations (ie, HER social app, Autostraddle, Lez Do Brunch, and the Los Angeles Lesbian, Gay, Bisexual, and Transgender [LGBT] Center) trusted as sources of health and social information by LBQ women. In addition to cultural tailoring, LezParlay draws upon the self-determination theory (SDT [77,78]) and the nascent gamification literature [79-82] to leverage 4 evidence-based game mechanics (ie, copresence, a system of points, user-generated content, and chance-based uncertainty) to both remedy the limitations associated with traditional PNF intervention formats and foster basic psychological needs for relatedness, competence, and autonomy in this population (see Multimedia Appendix 1 [83-102] for an overview of LezParlay game mechanics and supporting literature).

One round of LezParlay was played monthly over an 8-month period, with a variable cash prize awarded monthly to the top scoring player exhibiting the greatest accuracy in their perceptions of LBQ peers. During the first 3 weeks of each round, players were invited to size-up fellow LBQ players by browsing their social media–like profiles, submit guesses about negative stereotype-related behaviors and experiences of age- and sexual identity–matched LBQ peers (eg, What percentage of [lesbian/bisexual/queer] players in their [20s/30s/40s/50s+] own a pair of Birkenstocks? How many days per week does the typical [lesbian/bisexual/queer] player in her [20s/30s/40s/50s+] drink?), select an amount of points to wager on these guesses being true of other age- and sexual identity–matched players, and earn points for reporting on their own corresponding behaviors and experiences. In the last week of each month, all players received individualized, detailed results (ie, PNF) for a subset of the round’s questions. Animated charts and text detailed the accuracy of the player’s perceptions, how their behaviors and experiences compared with LBQ peers, summarized the stereotypes challenged, and provided their perceptual accuracy–based rank and score. Importantly, all actual norms featured in the detailed results were organically derived from the round-specific reports of players’ behaviors and experiences. Multimedia Appendix 1 provides additional literature supporting this innovative approach to the PNF intervention as well as detailed descriptions of the LezParlay round play and detailed results (ie, PNF screens).

### This Study

This registered clinical trial sought to evaluate whether LezParlay-delivered PNF on alcohol use reduces alcohol consumption and negative consequences relative to PNF on control topics (aim 1); examine whether providing PNF on coping behaviors, in addition to alcohol use, further reduces alcohol use and consequences beyond alcohol PNF alone (aim 2); identify mediators (ie, perceived norms) and moderators (ie, interpersonal stigma exposure, baseline drinking, sexual identity, age, relationship status, and race or ethnicity) of intervention efficacy (aim 3); and examine broader LezParlay competition engagement, acceptability, and perceived benefits (aim 4). The following sections provide an overview of the trial design, LezParlay competition promotion efforts, randomized controlled trial (RCT) subsample recruitment, measures, and analysis plan.

## Methods

### Trial Design

Following recent recommendations for testing the real-world feasibility and impact of normative feedback interventions [62,103], LezParlay was examined through a type 1 hybrid-effectiveness-implementation trial [104,105]. That is, in contrast to recruiting LBQ women into a transparent, incentivized, alcohol intervention study, LezParlay was advertised as it would be in the real world—as a free, web-based competition designed to test LBQ stereotypes and increase visibility. Only after several rounds of play were a subsample of 500 drinkers, already participating in the competition, invited to participate in an incentivized evaluation study. These players were covertly randomized to receive 1 of 3 unique sequences of feedback (ie, alcohol+coping, alcohol only, or control only) over 2 consecutive rounds of play. Short-term reductions in norms and drinking were assessed 2 months later organically within the competition through a *replay bonus*, which invited players to boost their scores by guessing, betting, and reporting on alcohol use and control topics a second time. Following the competition, 4 months postintervention, evaluation study participants then completed a feedback survey assessing competition acceptability, perceived benefits, and feature requests for the next version of the competition. At the end of this survey, participants reported their alcohol use one final time.

### Application Technology

LezParlay is a low-cost, device-responsive, HTML5 progressive web application integrated with Facebook Connect and Construct 3 game engine as well as a text message application programming interface, and email server. Although it provided a native app–like feel on Android and Apple smartphones, the app could be accessed on any internet-connected electronic device and did not require players to visit an app store or download any software. Instead, SMW simply accessed the LezParlay web app by URL [106] and were provided instructions for saving the web app to their computer’s desktop or smartphone’s home screen for easy access.

### Competition Promotion

The competition was open to all LBQ women aged 21 years or above, regardless of birth sex. Players learned about LezParlay through 1 of 4 promotion strategies taking place over a 3-month period. First, before the launch of the first round, local SMW were invited to sign up through flyers and promotional items distributed at LBQ community events in Los Angeles (ie, a weekend brunch for queer women organized by a community group Lez Do Brunch and a queer casino night jointly organized by the Los Angeles LGBT Center and the Los Angeles Women’s Network). Next, as the first round began, marketing campaigns on the HER social app, the leading dating or social app for LBQ women, invited users in their 3 largest markets (ie, Los Angeles, New York City, and Chicago) to LezParlay via push notifications and in-app advertisements. An advertisement was also placed in the electronic newsletter of Autostraddle, the leading independently owned news website for queer women. During the first 3 rounds of the competition, targeted campaigns on Facebook and Instagram (Facebook Inc) also advertised LezParlay to LBQ women residing in the United States. All recruitment materials were linked to LezParlay’s informational landing page [107]. This page presented an overview of the competition and provided a sign-up button that redirected interested women to view and accept the terms of service and privacy policy (basic consent for competition participation) before creating an account. The institutional review board of Loyola Marymount University approved all recruitment materials, procedures, and intervention materials (protocol number LMUIRB2018SU14).

### Procedure

After consenting to take part in the competition, users were prompted to link a valid mobile phone number to their account, and they could elect to log in with a unique email address and password combination or use their existing Facebook credentials. Next, users created their LezParlay public profile, which included a username of their choice and their sexual identity, age group, relationship status, and pronouns. Users also had the option of uploading a profile photo or Bitmoji to represent them; entering a brief textual self-description; and connecting their Facebook, Twitter, and/or Instagram accounts so that other players could learn about them. Following account creation, players were directed to a home screen that displayed a timer counting down to the close of the current round as well as buttons to play the current round, browse player profiles, submit and vote on the questions to be parlayed in future rounds, view round winners and leaderboards, edit public profile, and change account settings. The specifics of round play and the format of the detailed results (ie, PNF) delivered at the end of each round are detailed in Multimedia Appendix 1.

### RCT Subsample Recruitment

There was no upper limit on the number of SMW who could take part in LezParlay, and new players were accepted on a rolling basis throughout the competition. We aimed to recruit a minimum of 1200 LBQ women to sign up during the first 2 monthly rounds to ensure that meaningful and stable sexual identity– and age group–specific actual norms for drinking and coping behaviors could be delivered in intervention rounds 3 and 4. From this larger pool of players, 500 drinkers were recruited to participate in a LezParlay evaluation study (RCT) during the third month of play. Acting as baseline (T1) for the RCT, round 3 featured questions about alcohol use, stigma experiences, and a group of nonhealth-related control questions submitted by players. Upon submitting answers to alcohol-related questions in round 3, players were covertly screened for evaluation study eligibility based on their answers (ie, number of drinking days per week and peak drinks on a single day during the past 2 months) as well as their geolocation and the number of previous rounds played. Those who played at least one previous round, were in the United States, and reported drinking alcohol on 3 or more days per week or having 3 or more drinks on their peak drinking occasion were invited to take part in the evaluation study at the end of the round. Interested potential participants advanced to an informed consent screen that explained that the goal of the study was to evaluate the impact and format of detailed results received in LezParlay and gather player feedback to inform the next version of the competition. The information further detailed that participation in the evaluation study simply involved playing and viewing detailed results in subsequent rounds and completing a brief feedback survey at the end of the competition. Participants could earn up to US $40 in electronic gift cards of their choice to play subsequent rounds and complete the feedback survey. Those who checked a box indicating that they understood what the study participation entailed and desired to participate were welcomed into the study as LezParlay *official testers*.

### RCT Design, Randomization, and Debriefing 

The web app’s Qualtrics integration ensured that Qualtrics Research Suite’s automated randomizer, commonly used in RCTs evaluating psychosocial interventions [40,41,55], could be used to randomize evaluation study participants to a PNF condition at the point of study enrollment in round 3. Randomization determined the sequence of topics on which participants received detailed results across intervention rounds 3 and 4: alcohol+coping, alcohol+control, or control only. Members of the research team were blinded to participant condition assignment, and the study participants were not aware that any sort of randomization was taking place. Rather, when detailed results were sent at the end of each round, players were prompted to choose among 3 animated, graphical doors to determine the 1 to 2 round topics on which they would view detailed results (see Figure S2 in Multimedia Appendix 1). Although the results topics were truly determined via chance in most rounds of the competition, the doors of evaluation study participants were *fixed* to open to their randomly assigned feedback topics regardless of the door they selected in rounds 3 and 4. Upon completing the feedback survey at the end of the competition, participants were debriefed regarding the study’s research questions and the fixed sequences of health or control feedback they were randomized to receive in rounds 3 and 4 of the competition.

### Intervention Rounds

All players taking part in the third round of LezParlay estimated the drinking behaviors of the typical same-sexual identity player in their age group during the previous 2 months, reporting on their perceptions of the typical player’s (1) maximum number of drinks consumed on a single occasion, (2) average number of drinks consumed per occasion, and (3) average number of drinking days per week [103]. Players also estimated the number of negative alcohol-related consequences experienced over the previous 2 months by a typical player in their sexual identity and age group from a list of 8 negative consequences (ie, had a hangover or illness, got in a physical or verbal fight, had problems with significant others, missed a social engagement or event, had problems with friends or family, performed poorly at work or school, had problems with money, and had an unwanted or regrettable sexual experience). Players then answered parallel items assessing their own drinking and consequences over the corresponding 2-month period.

Players taking part in round 4 of the competition were prompted to think about how other players deal with stress and sexual minority stigma and asked to estimate the percentage of time (ie, 0%-100%) a typical player in their sexual identity and age group tried to feel better during the past month by (1) drinking alcohol; (2) taking a drug; (3) meditating, using relaxation techniques, or exercising; and (4) talking to a close other or mental health professional. Players were then prompted to think about how they themselves dealt with stress and stigma and responded to parallel items. The actual norms variably delivered to evaluation study participants in PNF at the end of rounds 3 and 4 were derived by computing the actual average response of all players submitting responses in each sexual identity and age group.

Participants randomized to receive PNF on control topics received detailed results for nonhealth-related topics in round 3 (eg, household repair ability, frequency of home improvement store visits, or tool box ownership) and round 4 (eg, time in between relationships, texting exes, and partners being confused for sisters). Multimedia Appendix 1 includes a link to view an example of detailed results for a control topic and a treatment topic delivered in the intervention rounds.

### RCT Measures

#### Demographic and Psychosocial Covariates

At sign up, all players reported their sexual identity, relationship status, and age group. Upon enrolling in the evaluation study, participants also reported their race, ethnicity, and actual age in years. The feedback survey at the end of the competition prompted the study participants to rereport their relationship status and sexual identity.

#### Perceived Alcohol-Related Norms and Behaviors 

As described previously, perceived drinking norms and alcohol-use behaviors were assessed organically in competition rounds 3 (T1; baseline) and 7 (T2; 2-month follow-up) by items modeled after Baer’s quantity, frequency, and max measure [108] in combination with additional norm and behavior items, respectively, examining negative alcohol-related consequences. These items were assessed a final time at the end of the postcompetition survey (T3; 4-month follow-up). Measures at each timepoint were referenced from the previous 2-month period. As done in previous gamified PNF pilot studies with college students [43,109,110], composite measures of perceived alcohol-use norms and alcohol-use behavior at baseline and follow-up will be computed by z scoring and then averaged across respective sets of individual items at each timepoint. In addition to these composites, 3 key outcomes of interest in alcohol intervention research are to be examined individually pre- and postintervention: (1) estimated drinks per week over the previous 2 months (computed by multiplying the reported number of drinking days per week and the average number of drinks per occasion at each timepoint), (2) peak drinks on 1 occasion over the previous 2 months, and (3) the number of negative alcohol-related consequences over the previous 2 months.

#### Interpersonal Stigma Exposure

Interpersonal stigma exposure was also assessed at baseline (round 3) and follow-up (as a replay bonus topic in round 7). Players guessed about the stigma experiences of other players and reported on their own stigma experiences over the previous 2 months. Stigma-related norms were not corrected in the competition, and these perceptions were only assessed to make sense for players to report on their own recent interpersonal stigma exposure (a theorized moderator of conditional effects on drinking) at the same time that they were reporting on their alcohol use and negative consequences in the game. Players’ recent exposure to severe interpersonal stigma was assessed by their responses to 2 items: (1) “During the past 2 months, how many times have you been physically harmed due to your sexual identity?” and (2) “During the past 2 months, how many times have you been verbally harassed or threatened (online or in person) due to your sexual identity?” Associations between pairs of stigma items at each timepoint are expected, and we anticipate combining responses to derive severe interpersonal stigma scores.

### Feasibility Measures

#### Reach and Engagement

Data from Google Analytics and the app’s back end will allow us to examine the total number of players who signed up to participate in the LezParlay competition in the absence of traditional study participation incentives; identify the promotional channels that brought them to the app; and detail the players’ demographic characteristics, states of residence, average number of log-ins, and number of rounds completed.

#### Acceptability

Feedback surveys prompted study participants to rate numerous aspects of the competition (the stereotype challenge concept, topics and questions, detailed results, leaderboards, the ability to browse player profiles, the ability to submit questions, the ability to bet points on the accuracy of guesses, text messages, and email communications from LezParlay) on Likert-type scales ranging from did not like at all (0) to liked very much (5).

#### Perceived Benefits

A single yes or no item asked participants whether they felt that participating in the LezParlay competition was psychologically beneficial. Those selecting *yes* in response were asked to enter text describing the perceived benefits.

#### Improvements and Requested Features

A final free-response item asked participants to share recommendations they had for improving the competition and describing the features they would like to see in the next version.

## Results

### Overview

This project was funded by the National Institute on Alcohol Abuse and Alcoholism in April 2018. The institutional review board approval was granted in August 2018, and the LezParlay app was developed per approved protocol between August 2018 and November 2018. The competition launched in January 2019, and the collection of all efficacy and feasibility data was completed in September 2019. A total of 2677 LBQ women participated in the LezParlay competition, with 500 LBQ drinkers recruited into the efficacy trial. Data cleaning and analysis were delayed by several months because of COVID-19–related delays and is underway as of January 2021. The results are expected to be published in summer 2021.

### Data Analytic Plan for Evaluating Intervention Efficacy

An intent-to-treat approach will be used to examine LezParlay treatment effects at 2 and 4 months postintervention on 4 outcomes: composite alcohol use, estimated number of drinks per week, peak number of drinks on 1 occasion, and number of negative alcohol-related consequences. Preliminary analyses will examine potential biases related to attrition and missing data [111,112], inspect outcome distributions, and evaluate potential baseline differences among conditions. As the latter 3 outcomes are count variables (ie, estimated drinks per week, peak drinks, and negative consequences), they are likely to be substantially skewed and best approximated by either Poisson or negative binomial distributions.

#### Main Effects

At 2 and 4 months after the delivery of treatment PNF, participants in both conditions receiving treatment PNF on alcohol use (ie, alcohol+coping and alcohol only) are expected to report reduced drinks per week, peak drinks, and negative consequences relative to those in the control PNF condition. Furthermore, participants in the alcohol+coping condition are expected to exhibit larger reductions in their alcohol use and negative consequences at postintervention follow-ups than participants in the alcohol-only PNF condition. Multilevel models (MLMs [113,114]) with full maximum likelihood specification will be used to test these predictions. Time will be specified as a level 1 varying predictor nested within individuals (level 2). Intercept treatment differences will represent treatment differences at baseline (eg, conditional differences in drinking at baseline), and slope differences will represent changes over time (eg, did participants in treatment conditions reduce their drinking between baseline and follow-up assessments more than control participants). The intercept includes a random effect, which will model the subject-specific heterogeneity in alcohol-related outcomes, thereby controlling for correlated data due to individuals. Main effect models will also control for covariates: age, sexual identity, race, ethnicity, relationship status, and severe interpersonal stigma exposure.

#### Tests of Mediation and Moderation

Tests of mediation will examine whether perceived drinking norms at the 2-month follow-up mediate relationships between condition and alcohol-use outcomes at the 4-month follow-up. PROCESS bootstrap tests [115,116] will be used to test the mediation. These models will control for baseline measures of potential mediating variables (ie, norms) and outcomes (ie, alcohol use and consequences). Moderation analyses will be examined within an MLM framework and will examine whether the efficacy of treatment PNF varied as a function of participants’ baseline drinking, sexual identity, exposure to severe interpersonal stigma, or other demographic characteristics. In the presence of significant interactions, exploratory moderated mediation models [115,117,118] may simultaneously estimate the conditional direct and indirect effects associated with the different levels of moderating variables.

#### Power Analysis

Informed by previous research examining the effects of web-based alcohol PNF on changes in normative perceptions and drinking in other populations (Cohen *d*=0.22 [20,119]), the comparably larger effect size revealed in a similar gamified PNF intervention for college students (Cohen *d*=0.46 [109]), power analyses using the standard 0.80 power of detecting a significant effect, *P*<.05, and an effect size of Cohen *d*=0.30 indicate a sample size of 375 (125 participants in each condition) to be sufficient to detect small-to-medium effects using repeated measures MLMs (ie, 2 levels, 3 arms, and randomization at the individual level) as well as tests of mediation and moderation. Thus, our sample size of 500 will allow us to detect modest effects with even 30% attrition.

### Data Analytic Plan for Evaluating Feasibility

Descriptive statistics will allow us to assess SMW’s level of interest in the LezParlay competition and engagement with the app (ie, total number of sign-ups and average number of log-ins), recruitment origins (eg, HER app ad, Facebook, Instagram, and player referral), acceptability (mean rating overall and by competition component), and perceived psychological benefits (ie, proportion of evaluation study participants who reported benefits). Qualitative text entry responses to items assessing the perceived benefits of the LezParlay competition and improvements or features requested for the next version will also be coded by theme or category using a generic inductive qualitative coding approach [120].

## Discussion

### Trial Overview

Joining the growing number of gamified health interventions being developed for sexual minority men and youth [121-126] and extending gamified PNF pilot work with college students [43,83,109,110], this project leverages evidence-based digital game mechanics informed by SDT to deliver PNF within a novel digital competition designed to challenge LBQ stereotypes and increase visibility. Notably, several deep-structure themes well documented among LBQ women are incorporated into the incognito intervention to bolster both relevance to and resilience within this population. For instance, awareness of stigmatizing sexual identity–based stereotypes is well documented among LBQ women [63-66], and social norms theory predicts that players are likely to overestimate undesirable stereotypical behaviors among peers of same-sexual identity (eg, promiscuity and infidelity among bisexual women, unhealthy relationship behaviors, and transphobic attitudes among lesbians). As such, we anticipate that revealing and reinforcing true norms for such experiences and attitudes may carry psychological benefits (ie, reducing identity-related stigma and increasing collective self-esteem) for participants beyond reduced alcohol consumption.

This hybrid trial also follows recent recommendations for improved design and evaluation of social norms–based health interventions [62], as both mediators and moderators of intervention effectiveness are examined, and, importantly, the LezParlay competition is framed and advertised as it would be outside of the study setting. Only later is an incentivized clinical trial subsample of drinkers recruited from the larger population of players already engaging with the competition (notably in the absence of traditional study participation incentives). This hybrid design allows the research team to cost-effectively assess the feasibility of drawing large numbers of SMW to the broader LezParlay competition via targeted promotional channels, player engagement with different areas of the web app, and competition acceptability and ways in which the competition might be improved. Simultaneously, recruiting a subsample of alcohol-consuming SMW already taking part in the competition into an incognito RCT allows for the evaluation of whether PNF on alcohol use and stigma-coping behaviors meaningfully reduces alcohol consumption and negative consequences relative to control PNF. In sum, this design allows critical questions about feasibility and efficacy to be jointly addressed, with minimal costs to internal or external validity.

The examination of demographics in terms of both LezParlay engagement and moderators of intervention efficacy will identify the groups of SMW most engaged and impacted by this strategy. Furthermore, as previous work with SMW has not examined potential interactions between perceptions of sexual identity–specific drinking norms and experiences with violence or harassment because of sexual minority status, examining the potential interplay between these established predictors of drinking allows this project to make a significant contribution to the larger sexual minority health literature. Similarly, as normative feedback interventions have not been widely considered as potential strategies for reducing problematic substance use or other health-risk behaviors among members of stigmatized health disparity populations, determining whether minority identity–based violence and harassment makes PNF more or less efficacious is a critical research question that may also carry intervention development implications for these populations (eg, racial and ethnic minorities and gender minorities).

### Limitations and Future Directions

Funded by the National Institutes of Health’s exploratory/developmental R21 grant mechanism, the goal of this initial trial is to evaluate the feasibility and efficacy associated with the LezParlay competition as a *minimally viable product* taking the form of an extremely low-cost, progressive web app designed and coded by the first author, who is a member of the target population. Thus, representing the preliminary step in a larger program of LezParlay-gamified PNF intervention research, findings from this trial will not speak to the feasibility or efficacy of delivering the intervention through a more sophisticated and polished native smartphone app that would likely be more desirable and user-friendly and thus better equipped to attract and retain LBQ women. In addition, PNF is the only intervention component featured in the initial version of LezParlay and, furthermore, only static descriptive norms for drinking and coping are corrected. Findings from this study may suggest high feasibility for the gamified approach and stereotype challenge framing (ie, large numbers of SMW are engaged by the competition and participants and report psychological benefits), but treatment PNF fails to meaningfully reduce drinkers’ consumption and negative alcohol-related consequences relative to control. In this event, the stereotype challenge concept and game mechanics may be retained; however, future versions of the LezParlay app might expand PNF to correct additional types of alcohol and coping norms (ie, injunctive, affective, and dynamic norms) and/or deliver additional intervention components such as skills training around healthy coping strategies and/or local alcohol treatment information (ie, referral to treatment).

The intervention’s real-time, organic generation of actual norms from LezParlay players’ round-specific reports of their behaviors represents both a point of innovation and a potential limitation. Foremost, this approach diverges significantly from traditional PNF interventions that derive actual drinking norms from an existing data source or a separate norms documentation study conducted before the recruitment of the intervention sample [19-22]. As PNF research suggests that the practice of using previously collected actual norms data may undermine intervention efficacy by diminishing PNF credibility and interest [55-57,110], LezParlay sought to increase the psychological proximity and relatability of the peer group by making the individuals on whom actual norms are based digitally present and visible. As a point of innovation, this format may help extend PNF interventions to sexual minorities and other hard-to-reach populations for whom existing norms data do not exist and would be difficult and/or costly to collect through a separate survey study. Furthermore, trial results from a similar gamified PNF intervention for college students revealed that the actual drinking norms similarly derived in-game among visible peers differed very little from those derived from separate survey samples of students at the same university [109]. However, as this approach has not previously been tested with adult LBQ women, it is critical to establish that the actual norms derived in real time from LezParlay rounds are sufficiently risk-reducing, stable, and approximately equivalent to those derived from comparable survey samples containing the same age and sexual identity groups [14-16,91].

Another limitation pertains to this study’s organic assessment of alcohol outcomes and some moderators within the rounds of the competition. Although this is a major strength, in that it eliminates the demand characteristics that often plague transparent alcohol intervention studies and substantially decreases trial costs, this also meant that the key constructs could only be assessed by a few items, and the language of the items could not be too formal or clinical in tone. Although Baer’s frequency, quantity, and max measure [103] fit well in this regard as a short, validated measure of alcohol use, it would have also been valuable to include longer validated survey measures to more formally assess alcohol-related outcomes and screen for alcohol use disorder. In the event that LezParlay feasibility and efficacy are demonstrated in the initial trial, we anticipate seeking additional funding for a larger trial that will include traditional survey-based baseline and follow-up assessments and test an expanded set of potential mediators and moderators.

A final limitation pertains to this study’s narrow focus on the direct effect of PNF treatment on alcohol-related outcomes. Norms for other health behaviors (ie, stigma coping, smoking, exercise, and health care utilization) were also corrected within the broader competition; however, the RCT was not designed to examine potential PNF-related changes in these behaviors. Similarly, players were expected to overestimate several undesirable stereotypical behaviors among same-sexual identity peers in nonintervention rounds of the competition (eg, promiscuity and infidelity among bisexual women, unhealthy relationship behaviors, and transphobic attitudes among lesbians). Although outside the scope of this initial trial, revealing and reinforcing true norms for these experiences and attitudes in LezParlay may carry psychological benefits for SMW partaking in the competition (ie, reducing identity-related stigma, increasing feelings of belonging, and/or collective self-esteem). Thus, assessing pre-post competition changes in these constructs and evaluating the extent to which challenging negative stereotypes through the larger LezParlay competition might buffer stigma-related processes and thereby reducing drinking and improving other health outcomes remain to be critical next steps in the larger program of research.

### Conclusions

This hybrid trial will examine the efficacy and feasibility of an innovative, culturally tailored, evidence-based alcohol intervention for LBQ moderate-to-heavy drinkers, thereby narrowing the disparity in alcohol intervention research and practice. This *incognito*, gamified intervention approach is uniquely oriented toward engaging and preventing alcohol-related risks among LBQ community members who may view their heavy drinking as normative and not in need of change because of the visibility of alcohol consumption in LBQ community spaces. Thus, this intervention strategy diverges from and is intended to complement more intensive intervention programs being developed to meet the needs of SMW already motivated to reduce their consumption and those seeking culturally tailored treatment for alcohol-use disorder and comorbid mental health problems [40,41].

## Appendix

Multimedia Appendix 1 Overview of LezParlay game mechanics, supporting literature, round play, and detailed results.

Multimedia Appendix 2 Peer-review report by the National Institute on Alcohol Abuse and Alcoholism.

## Abbreviations

LBQ: lesbian, bisexual, or queer
LGBT: lesbian, gay, bisexual, and transgender
MLM: multilevel model
PNF: personalized normative feedback
RCT: randomized controlled trial
SDT: self-determination theory
SMW: sexual minority women
