# Supplementary material for: A Gamified, Social Media–Inspired, Web-Based Personalized Normative Feedback Alcohol Intervention for Lesbian, Bisexual, and Queer-Identified Women: Protocol for a Hybrid Trial
Source: JMIR Res Protoc. 2021 Apr 16;10(4):e24647. doi: 10.2196/24647 (PMC8087973; doi:10.2196/24647)
Supplement: Multimedia Appendix 1 [file resprot_v10i4e24647_app1.docx]

**Multimedia Appendix**

**LezParlay Competition Game Mechanics & Supporting Literature**


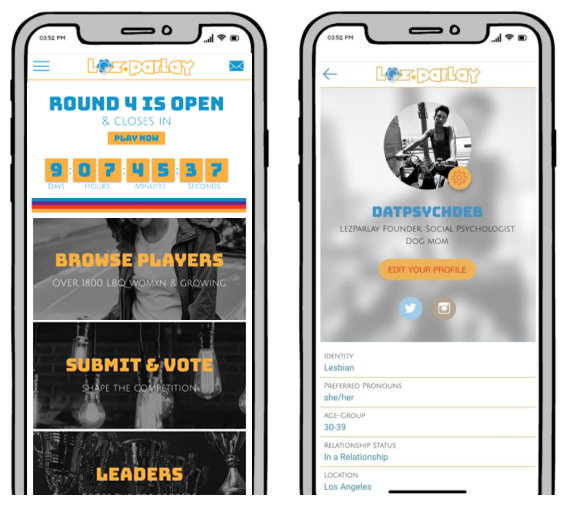


Figure 1. LezParlay app home screen and user profile

***Social Media Inspired Copresence & Real-Time Norm Generation.***

Within self-determination theory (SDT), relatedness is a psychological need satisfied when a person feels connected to and familiar with relevant others while taking part in an activity [74,108,109]. Further, in digital gaming contexts knowledge about others competing increases players’ interest, enjoyment, and the degree to which they find meaning through play [110-113]. However, in traditional web-based PNF interventions, knowledge about the peers in question is quite low, as is relatedness. Perception questions abstractly inquire about the behaviors of a typical member of the participant’s peer group and the actual norms delivered in PNF are derived from some previously collected, peer-group convenience sample [19,22]. As little is known about these peers, researchers have long speculated that such use of previously collected data may limit intervention efficacy by diminishing PNF credibility, interest, and attention [55-57]. This possibility was tested directly in a recent experimental PNF study with college students. With actual norms derived from all participants and held constant across conditions, a web-based gamified intervention condition which inquired about the behaviors of fellow digitally present and visible peers and framed actual norms as having been generated by the responses of these peers produced larger reductions in drinking than did the traditional PNF condition which more abstractly inquired about the behaviors of non-present peers and framed actual norms as being based on a previously collected survey data [90]. Subsequently, real-time norms generation among visible peers was featured in a real-world trial of a web-based gamified PNF intervention for college students with treatment arms found to be highly effective [89].

Building on this work, LBQ women taking part in LezParlay were able to create social media-like personal profiles, display avatars, and browse the profiles of fellow players in order to inform their guesses about behaviors and experiences of peers. Actual norms included in PNF were then transparently generated from the responses of players completing each round. This social-media inspired copresence was intended to both make PNF more impactful as suggested by work with college students [89,90] and increase lesbian, bisexual, and queer female visibility—something widely desired in this population [67,68,114]. As research with lesbian-identified SMW suggest that greater social media involvement is associated with heavier drinking, inflated perceptions of peer drinking norms, and greater confidence in normative misperceptions [115], LezParlay’s social media-inspired copresence features also aimed to both attract and maximally benefit heavy social media using LBQ women.

***Multiple Topics, User-generated Questions, & Chance-based Uncertainty.***

Autonomy within SDT concerns a sense of volition or willingness during a task and digital games that appeal to user’s social identities, interests, and values [116-120] and those that offer players greater choice over the course of play tend to increase autonomy and motivation [121,122]. LezParlay not only offerred autonomy through it’s packaging of a traditionally dry alcohol intervention strategy within a fun, social identity-relevant competition for LBQ women, but additional autonomy inducing features were leveraged in the competition to mitigate non-treatment seeking drinkers’ psychological reactance and defensiveness when viewing alcohol PNF [56,57]. Pilot work with college students has established that these negative reactions can be significantly reduced by providing PNF on multiple topics of interest to the target population and by giving alcohol-related PNF the appearance of being selected by chance [43,107]. Further, a previous trial investigating a multi-round gamified PNF competition for college students revealed that including alcohol-related questions in fewer than half of the total rounds played ensured that participants did not view the activity as an intervention intended to reduce their drinking [89]. As such, alcohol use appeared to be selected by chance among other topics in 2 of LezParlay’s 8 total rounds. Non-intervention topics were designed to be high interest and relevance to this population (e.g., stereotypes related to sex, relationships, style, hobbies, etc.) and players were also invited to help shape the competition by submitting questions to be “parlayed” in future rounds and voting on their favorites submitted by other players. Each of LezParlay’s rounds featured several top-voted user-generated questions and began with an animated spinners that appeared to determine the round’s topics and amount of the round’s prize. As digital game researchers have theorized that the placement of chance elements immediately before intervention content may facilitate the cognitive re-framing of the content, giving it a positive and serendipitous feel [123-124], at the end of each round players also selected between doors to unlock detailed results (i.e., PNF) on specific topics.


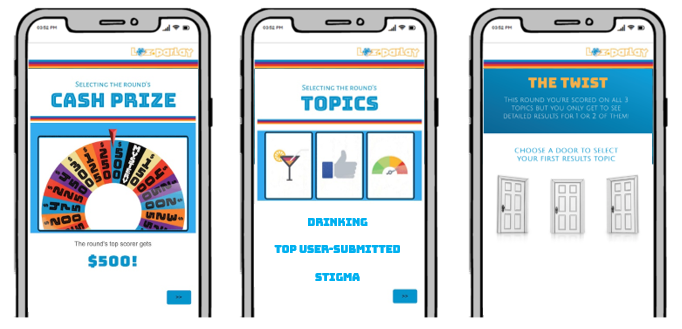


Figure 2. LezParlay chance-based uncertainty game mechanics

***System of Points***. In SDT, competence is a psychological need encompassing the desire for challenge as well as for feelings of capability and growth [74,75]. Satisfaction of this need through frequent rewards, feedback, the ability to track performance over time, and compare performance with others is associated with greater engagement sustained digital game play [120,124-126]. To satisfy competence needs, LezParlay invited SMW to guess about the behaviors and experiences of the typical player sharing their sexual identity and age-group, wager points on the degree to which they think their guess was correct based on the answers submitted by these players, and earn bonus points for reporting on their own parallel behaviors and experiences. Points were won and lost in LezParlay based on the accuracy of guesses and sizes of wagers. Leaderboards within the app displayed the Top 100 scorers following each round of play and variable cash prizes were awarded to the top monthly and cumulative scorers. Further, select rounds featured a “Replay Bonus” which allowed players to re-play a topic they previously received PNF on, thereby boosting their score by demonstrating increased competence.


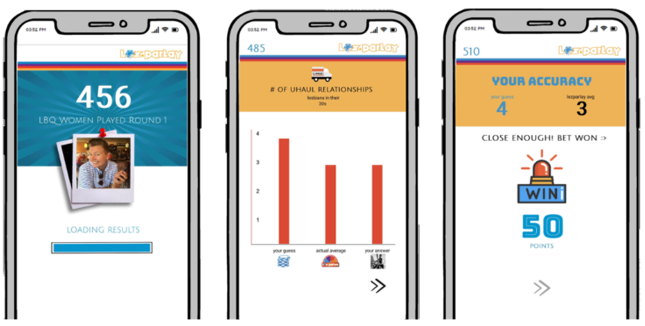


Figure 3. LezParlay detailed results with wager-based scoring

**LezParlay Competition Overview: Round Play and Delivery of Detailed Results**

***Round Play.*** Each round began with a “parlay period” which spanned the first 3 weeks of the month. Players could login to lock in their guesses about other players, set their point wagers, and report on their own parallel behaviors and experiences at any point during this period. Each round was designed to take no longer than 6 minutes to complete and included 2-3 topics ostensibly selected by a slot machine type spinner on an initial screen. Following topic selection, a wheel of fortune type spinner then selected the amount of the round’s top scorer cash prize (prizes ranged from $50 to $500). For each topic, 3-4 questions prompted players to guess about the behaviors and experiences of other players sharing their sexual identity and age-group (e.g., “How many “uhaul” or cohabitating relationships has the typical lesbian in her 30s had?) and select a number of points to wager (i.e., 10, 25, 50, 100) on this guess being correct based on the actual, average response of players in her sexual identity and age-group. Next, players reported on their own behaviors and experiences by answering parallel items (e.g., “How many “uhaul” relationships have you had?”) and earned 25 bonus points for each response. At the end of the 3-week period, the round closed and the numbers were manually crunched. For each question, the average response from players in each age and sexual identity group regarding their behaviors and experiences constituted the correct answer to be featured in detailed results (i.e., the actual norm in PNF) at the end of the month, with point wagers won and lost accordingly. The topics assessed and cash prizes awarded in each round of the competition are presented in Table 1.

Table 1.

*Summary of question topics and prizes awarded across rounds of the LezParlay competition.*

| Round # | Top-Scorer Prize | Question Topics |
| --- | --- | --- |
| 1 | $200 | Pets & Uhauling |
| 2 | $300 | Style & Identity Experiences |
| **3** | **$500** | **User-Submitted, Drinking, & Stigma** |
| **4** | **$400** | **User-Submitted, Coping, & Relationships** |
| 5 | $200 | User-Submitted & Infidelity |
| 6 | $300 | User-Submitted, Politics, & Coming Out |
| **7** | **$500** | **Drinking & Stigma (REPLAY BONUSES), Sex** |
| 8 | $400 | Uhauling & Pets (REPLAY BONUSES), Health |

*Note.* Bolded rounds 3, 4, and 7 were critical to the efficacy study. Condition assignment determined whether PNF on treatment or control topics were delivered at the end of Rounds 3 and 4.

***Detailed Results (PNF).*** Construct 3 game engine was programmed to create a private URLs presenting fun, animated, personalized detailed results for each player. Players completing each round were text messaged and emailed their private results links at the end of each month. Upon opening this link on any web-connected device, an initial screen detailed the player’s total score and wagers for the round. Next, the total the total number of players who completed the round thereby contributing to correct answers (i.e., actual norms) and an animated reel of avatars representing these players quickly flashed across the screen. Subsequent screens prompted players to select doors in order to reveal the topics on which they would view detailed results (see Figure 2). Then, results for each question were presented with an initial screen reiterating the question. Consistent with PNF [17,19,22], the second screen displayed an animated, horizontal bar chart with 3 bars contrasting: 1) the player’s guess about the answer of other players’ in her age and sexual identity group (perceived descriptive norm); 2) the correct answer based on the actual responses of players in her age and sexual identity group (actual norm); and, 3) the players own answer (own behavior). Following this bar chart, arcade like animations and sound effects revealed the player’s win or loss of points based on the accuracy of her guess and point wager. After these screens were displayed for each question within a topic, a final screen presented a chart revealing how the player’s behavior for the topic compared to other players across age-groups and sexual identities before moving on to the next topic. Following all topic-specific screens, a summary screen highlighted relevant stats that shattered or confirmed stereotypes related to the round’s topics and a final stats screen provided an overview of the player’s round performance in terms of total score and overall rank among all players. The stats screen contained buttons allowing players to view the top 100 scorers in a leaderboard and begin the next round.

Example detailed results on one non-health-related control topic (user-submitted) and one treatment topic (alcohol use) can be viewed from any Wi-Fi-connected Apple or Android smartphone at the URL provided in the reference section [86]. Table 2 provides the actual norms derived from all players taking part in intervention rounds 3 and 4. It is important to note that although we had anticipated differences in actual norms by sexual identity and such differences emerged in a number of non-intervention rounds, sexual identity differences did not emerge for any of the intervention round items. As such, actual norms in intervention rounds were differentially presented in PNF to LBQ evaluation study participants based on age-group only.

Table 2.

*Competition derived actual norms for intervention topics by age-group*

|  | Age-Group | | | | | | | | |
| --- | --- | --- | --- | --- | --- | --- | --- | --- | --- |
|  | 21-29 years | | | 30-39 years | | | 40 years+ | | |
| **Round 3- Alcohol Use Actual Norms** |  | *n*=627 |  |  | *n*=498 |  |  | *n*=212 |  |
| Drinking days per week |  | 2 |  |  | 2 |  |  | 1.5 |  |
| Average drinks per occasion |  | 2.5 |  |  | 2 |  |  | 2 |  |
| Peak drinks on one occasion |  | 4 |  |  | 3 |  |  | 3 |  |
| Number of Negative Consequences |  | 2 |  |  | 1.5 |  |  | 1 |  |
|  |  |  |  |  |  |  |  |  |  |
| **Round 4- Coping Actual Norms** |  | *n*=503 |  |  | *n*=414 |  |  | *n*=186 |  |
| % of time drink alcohol to cope |  | 18 |  |  | 17 |  |  | 16 |  |
| % of time use drug to cope |  | 12 |  |  | 9 |  |  | 9 |  |
| % of time exercise, meditate, to cope |  | 55 |  |  | 61 |  |  | 49 |  |
| % of time seek social support to cope |  | 53 |  |  | 50 |  |  | 62 |  |
