# Supplementary material for: A Gamified, Social Media–Inspired, Web-Based Personalized Normative Feedback Alcohol Intervention for Lesbian, Bisexual, and Queer-Identified Women: Protocol for a Hybrid Trial
Source: JMIR Res Protoc. 2021 Apr 16;10(4):e24647. doi: 10.2196/24647 (PMC8087973; doi:10.2196/24647)
Supplement: Multimedia Appendix 2 [file resprot_v10i4e24647_app2.pdf]

SUMMARY STATEMENT
(Privileged Communication)

PROGRAM CONTACT:
Dr. Robert Freeman
301443-8820
rfreeman@mail.nih.gov

Release Date: 07/07/2017
Revised Date:

Application Number: 1 R21 AA025767-01A1

Principal Investigator

LABRIE, JOSEPH W

Applicant Organization: LOYOLA MARYMOUNT UNIVERSITY

Review Group: AA-2
Epidemiology, Prevention and Behavior Research Review Subcommittee

Meeting Date: 06/12/2017
Council: OCT 2017
Requested Start: 12/01/2017

RFA/PA: PA14-188
PCC: AP FD

Project Title: PNF 2.0: A Novel, Gamified, Facebook-Integrated Personalized Normative Feedback Intervention to Reduce Alcohol Use and Negative Consequences among Sexual Minority Women
SRG Action: Impact Score:19
Next Steps: Visit https://grants.nih.gov/grants/next_steps.htm
Human Subjects: 44-Human subjects involved - SRG concerns
Animal Subjects: 10-No live vertebrate animals involved for competing appl.
Gender: 2A-Only women, scientifically acceptable
Minority: 1A-Minorities and non-minorities, scientifically acceptable
Children: 3A-No children included, scientifically acceptable
Clinical Research - not NIH-defined Phase III Trial

Project Year	Direct Costs Requested	Estimated Total Cost
1	150,000	211,101
2	125,000	175,917
TOTAL	275,000	387,018

ADMINISTRATIVE BUDGET NOTE: The budget shown is the requested budget and has not been adjusted to reflect any recommendations made by reviewers. If an award is planned, the costs will be calculated by Institute grants management staff based on the recommendations outlined below in the COMMITTEE BUDGET RECOMMENDATIONS section.

1R21AA025767-01A1 LABRIE, JOSEPH

PROTECTION OF HUMAN SUBJECTS UNACCEPTABLE

RESUME AND SUMMARY OF DISCUSSION: This is an amended R21 application to test a culturally tailored, newly developed Gamified Alcohol Norm Discovery and Readjustment (GANDR) personalized normative feedback (PNF) intervention to narrow the disparity in alcohol misuse prevention among sexual minority women. The revised submission retains previously noted strengths - high significance of the proposal, a strong theoretical foundation, a strong investigative team with relevant expertise, social media recruitment via Facebook of sexual minority women, and the overall overall strong study design. The applicant has satisfactorily addressed most prior concerns. The few lingering concerns are related to recruitment, and were deemed minor and easily fixable. Overall, there was outstanding enthusiasm for this innovative application.

DESCRIPTION (provided by applicant): Sexual minority women in the United States are more likely to drink alcohol, engage in heavy drinking, and experience alcohol-related problems than are heterosexual women. Yet, to date, no evidence-based intervention or prevention efforts have been developed to reduce alcohol consumption in female sexual minority community settings. The proposed research seeks to narrow the disparity in alcohol intervention research by examining an innovative gamified personalized normative feedback (PNF) intervention to reduce drinking among members of a local sexual minority women community found to frequent Facebook and overestimate norms related to peers' general alcohol use and drinking to cope with sexual minority stigma. Our newly developed GANDR (Gamified Alcohol Norm Discovery and Readjustment) PNF format takes the well- established core components of a PNF alcohol intervention and delivers these components within an inviting, Facebook-connected, social game. This intervention format is designed to be more appealing, engaging, believable, positively received, and thus, effective than standard web-based PNF and can be culturally tailored to appeal to a number of different populations, including those typically hard to recruit into alcohol interventions. GANDR LA, the version developed for sexual minority women residing in Los Angeles County, delivers PNF on alcohol use and stigma-coping behaviors within the context of an online game about sexual minority stereotypes which incorporates users' Facebook photos to increase believability and a point-based reward system to increase motivation and engagement. In addition, to decrease defensive reactions and increase appeal, feedback topics are ostensibly selected by chance in GANDR LA with treatment PNF on alcohol use and stigma-coping behaviors provided alongside feedback on control topics of high interest to community members (e.g., activism, relationships, etc.). Additional appeal and credibility are gained through GANDR LA's sponsorship and promotion by a collaborating sexual minority community non-profit organization viewed as a trusted source for health and social information by members of the target community. After documenting sexual minority community norms (AIM 1) through an initial round of play (N=1275), a sub-sample of 675 sexual minority female drinkers will be randomized to receive 1 of 3 unique sequences of feedback (i.e., Alcohol & Stigma-Coping, Alcohol & Control, or Control topics only) during 3 intervention rounds taking place over a 6-month period. The randomized feedback sequences and multiple rounds of play will allow us to determine whether PNF on alcohol use corrects drinking norms, and reduces sexual minority women's alcohol consumption and negative consequences relative to PNF on control topics (AIM 2: H1), examine whether providing PNF on stigma-coping behaviors in addition to alcohol use further reduces alcohol use and consequences beyond standard alcohol PNF (AIM 2: H2), and identify mediators and moderators of intervention effectiveness (AIM 3).

PUBLIC HEALTH RELEVANCE

The proposed project partners an expert research team with a trusted sexual minority community non-profit organization to deliver an innovative gamified web-based personalized normative feedback (PNF) alcohol and stigma-coping intervention to local sexual minority women, thus narrowing the disparity in alcohol intervention research and practice. Specifically, this research implements and evaluates the

ability of a novel, Facebook-integrated PNF intervention disguised as an online game about harmful sexual minority female stereotypes to reduce sexual minority women's' alcohol use and negative consequences over a 6-month period.

CRITIQUE 1

Significance: 1
Investigator(s): 1
Innovation: 1
Approach: 1
Environment: 1

Overall Impact:

The investigator of this Exploratory/Developmental Grant Application (R21) seeks to narrow the disparity in alcohol misuse prevention among sexual minority women by testing a culturally tailored and newly developed Gamified Alcohol Norm Discovery and Readjustment (GANDR) personalized normative feedback (PNF) intervention. Several strengths were noted in the original application, including high significance of the proposal, a strong theoretical foundation, a strong investigative team with relevant expertise, social media recruitment via Facebook of sexual minority women, and an overall strong study design. The weaknesses included (a) A single item measure for coping; (b) The availability of the feedback on the screen for only 20 seconds; and (c) a lack of information on the feasibility of the ambitious recruitment strategy using Facebook. The strengths of the application have been maintained and the primary concerns have been addressed. The only small lingering concern is related to recruitment, but this is a minor concern.

1. Significance:

Strengths

- The proposed PNF intervention combines strong theoretical and substantive knowledge about lesbian bar culture and minority stress, to explain coping motivated drinking and the need for interventions.

Weaknesses

- None noted.

2. Investigator(s):

Strengths

- This is a very productive and strong investigative team experienced in PNF intervention design and evaluation, as well as research and intervention development with the target population.
- The coping skill research experience of the team has been highlighted and is sufficient to conduct the proposed research.

Weaknesses

- None noted.

3. Innovation:

Strengths

- The use of It use gamification to increase attention and engagement in the context of a PNF intervention among sexual minority, specifically lesbian, drinkers is highly innovative.
- Integrating coping norms to understand both motivations to drink and opportunities for intervention in this population is also innovative.

Weaknesses

- None noted.

4. Approach:

Strengths

- Overall the study is well designed and scientifically sound.
- Strong CBPR foundation and buy-in for the target population.
- The investigators have substantially refined the coping component of the intervention based on their recently published findings and the results from a new pilot study. Specifically, they found sexual minority adults, (and lesbian women in particular) to substantially over-estimate both the proportion of peers' who use alcohol to cope with stressors related to sexual minority stigma (i.e., prejudice, discrimination) and the quantity of alcohol consumed by peers in response to these stressors. They propose that prevalence and quantity norms related to drinking to cope with sexual minority stigma be corrected in the intervention, along with additional items correcting under-estimated adaptive stigma-coping norms.

Weaknesses

- None noted.

5. Environment:

Strengths

- The environment of Loyola Marymount University is appropriate to conduct this proposed research.

Weaknesses

- None.

Protections for Human Subjects:

Acceptable Risks and/or Adequate Protections

- Appropriate protections are in place for human subjects.

Data and Safety Monitoring Plan (Applicable for Clinical Trials Only):

Acceptable

- Data protections are adequate.

Inclusion of Women, Minorities and Children:

- Sex/Gender: Distribution justified scientifically
- Race/Ethnicity: Distribution justified scientifically
- For NIH-Defined Phase III trials, Plans for valid design and analysis: Not applicable

- Inclusion/Exclusion of Children under 18: Excluding ages <18; justified scientifically
- Distributions reflect the target population in the catchment area and are scientifically justified.

Vertebrate Animals:
Not Applicable (No Vertebrate Animals)

Biohazards:
Not Applicable (No Biohazards)

Applications from Foreign Organizations:
Not Applicable (No Foreign Organizations)

Select Agents:
Not Applicable (No Select Agents)

Resource Sharing Plans:
Acceptable

Authentication of Key Biological and/or Chemical Resources:
Not Applicable (No Relevant Resources)

Budget and Period of Support:
Recommend as Requested

CRITIQUE 2

Significance: 1
Investigator(s): 1
Innovation: 1
Approach: 2
Environment: 1

Overall Impact
This is a revised R21 application that proposes to narrow the disparity in alcohol intervention research by adapting and extending a personalized normative feedback (PNF) intervention strategy to reduce drinking among members of a local lesbian community in Los Angeles. Added literature, much from this research group, indicates that lesbians are found to both overestimate peer alcohol use norms and report frequent use of Facebook. In addition, it appears that coping with sexual minority stigma may influence drinking, and therefore reducing perceived norms of use as well as how many fellow lesbians drink to cope with stigma may reduce personal alcohol use and consequences. The research team will recruit 1275 self-identified lesbians ages 21-40 in three age cohorts to establish peer norm estimates, which will then be incorporated in their recently developed GANDR (Gamified Alcohol Norm Discovery

and Readjustment) PNF intervention and deliver its components within a Facebook-connected social game. Their research strategy will consist of large scale surveys from which to derive meaningful norms as well as a randomized trial with a subset of self-identified drinkers (N = 675, 225 in each age group) comparing three conditions: Alcohol + Coping topics, Alcohol +Control topics, or Control topics only. Participants will compete baseline, 1 and 6 month follow-ups. Despite some methodological concerns, this is a very thorough revision that addresses a significant issue in an innovative way.

1. Significance:
Strengths

- Lesbians drink more and experience more consequences than heterosexual women, and peer norms and coping may be significant contributing factors
- Personalized normative feedback interventions have reduced alcohol use and consequences in other populations, but none have been developed for lesbians
- Facebook is an appropriate modality to deliver a PNF intervention to the lesbian community
- Successful completion of the proposed aims may very well change PNF technologies and preventative interventions in the L (and GBT) community
- The investigators have clarified how alcohol use in lesbian population, albeit more than that reported in heterosexual women, takes a significant personal and societal toll, and also provided pilot data and recently published research to strengthen the relevance of stigma and coping motives
- Examination of alcohol use and consequences broadens impact and relevance of research

Weaknesses

- None noted

2. Investigator(s):
Strengths

- Dr. LaBrie is an accomplished researcher in the field of PNF and lesbian health and has conducted several R21 projects
- Dr. Costine is an appropriate consultant and her experience will greatly enhance the legitimacy and “buy-in” of the proposed project by the LA lesbian community and also provide a unique perspective to the interpretation and dissemination of the findings.
- Dr. Boyle is an ideal project director and has done impressive work in the field with the Co-Is, and has recently published relevant studies on the topic.
- Dr. Omoto is a leader in LGBT health and the PI of several federally funded research projects in the area.

Weaknesses

- None noted

3. Innovation:

Strengths

- Very innovative use of Facebook to recruit and assess members of the lesbian community in LA, especially the use of photos and the “slot machine” style of “randomly” presenting PNF on a variety of topics to reduce defensiveness to feedback

- Important and thoughtful strategy to shift current research or clinical practice paradigms by utilizing Facebook to collect data re: norms and deliver a targeted intervention.

Weaknesses

- None noted

4. Approach:
Strengths

- The overall strategy, methodology, and analyses are thoughtful, well-reasoned, and appropriate to accomplish the specific aims of the project
- Potential issues are described and reasonably addressed
- Pilot data from the GANDR format with lesbians as well as other populations (e.g., college students) on Facebook Integration and Gamification is a valuable addition to the proposal
- Project is ambitious but feasible with this research team.
- 3 waves of data collection over 6 months a plus
- Addition of alcohol-related consequences as an outcome a valuable addition
- 3 group design seems adequately powered

Weaknesses

- This is an ambitious recruitment project – 2000 lesbians on email list, but not clear the percentage of whom were over 40 or under 21. If this is a significant number, the authors are going to have to resort to secondary recruitment strategies (e.g., flyers, Facebook ads) which may be problematic
- Not clear why there is no minimum threshold for “heavier” drinking (e.g., 1 heavy episodic drinking episode in past 2 weeks, even 3 or more drinks per occasion) for the “drinkers” in the study to enhance the likelihood of observing intervention effects
- Repeated measures ANOVA may not be the strongest analytic approach for this design

5. Environment:
Strengths

- The university and environment seem ideally suited to conduct this type of research.
- Data security measures are impressive
- The research team has excellent ties to the LA lesbian community

Weaknesses

- None noted

Protections for Human Subjects:

Unacceptable Risks and/or Inadequate Protections

- Data security measures are impressive, but clarification is needed regarding the use of Facebook user IDs, and the use of pictures as part of the intervention.

Data and Safety Monitoring Plan (Applicable for Clinical Trials Only):

Acceptable

- Certificate of confidentiality to be obtained for the study

Inclusion of Women, Minorities and Children:

- Sex/Gender: Distribution justified scientifically
- Race/Ethnicity: Distribution justified scientifically
- For NIH-Defined Phase III trials, Plans for valid design and analysis: Scientifically acceptable
- Inclusion/Exclusion of Children under 18: Including ages <18; justified scientifically
- The proposal seeks to recruit lesbian-identified women, ranging in age from 21 to 40 years and residing in Los Angeles County: thus, 100% of the participants.
- The racial and ethnic breakdown of the L-Project LA's email list as well as Los Angeles County lesbians ages 21-40 who completed the California Health Interview Survey (CHIS) between the years of 2010 and 2014 were employed to project the demographic characteristics of participants in the proposed research. This data suggests that they can anticipate recruiting 680 White, 64 Asian, 172 African American, 192 Native Hawaiian or Pacific Islander, 1 American Indian/Alaska Native participant, and 166 participants of more than one race into the study. Of these participants, we expect that 491 will be of Hispanic ethnicity, with the majority of Hispanic participants being "White" or "More than one race".

Vertebrate Animals:

Not Applicable (No Vertebrate Animals)

Biohazards:

Not Applicable (No Biohazards)

Resubmission:

- The authors have done a thorough job addressing the previous reviewers' concerns./a

Applications from Foreign Organizations:

Not Applicable (No Foreign Organizations)

Select Agents:

Not Applicable (No Select Agents)

Resource Sharing Plans:

Acceptable

- The PI will adhere to the NIH Grant Policy on Sharing of Unique Research Resources, including the Sharing of Biomedical Research Resources Principles and Guidelines for Recipients of NIH Grants and Contracts. Requests for research resources that are generated as part of this project will be distributed in a timely manner. Distribution to academic and other non-profit researchers will occur under agreements no more restrictive than the Simple Letter Agreement for the Transfer of Materials as described in the Guidelines for Disseminating Research Resources Arising Out of NIH-Funded Research (Federal Register, Vol. 64, No 246, p.72094).

Distribution of resources to for-profit institutions will be determined based on the resource itself. As a means of sharing knowledge, the PI, who has a strong track record of dissemination with over 140 published peer-reviewed journal articles, will ensure timely publication of research findings in scientific journals, with acknowledgement that the research was supported by the NIH. These articles will also be submitted to PubMed Central (PMC). After publication of the main findings by the PI and project research team, the PI agrees to make copies of the final dataset available to other researchers upon request.

Authentication of Key Biological and/or Chemical Resources:

Not Applicable (No Relevant Resources)

Budget and Period of Support:

Recommend as Requested

Additional Comments to Applicant (Optional):

- Excellent job revising the proposal.

CRITIQUE 3

Significance: 3
Investigator(s): 2
Innovation: 2
Approach: 4
Environment: 1

Overall Impact:

While research has identified lesbian women as one of the heaviest drinking female demographic populations in the US, few of the alcohol intervention development efforts described in the literature have specifically focused on lesbian communities. This proposed intervention seeks to address this significant gap. The research team, with extensive background and experience in **personal narrative feedback** (PNF) intervention, based in Social Norms Theory, proposes to test the efficacy of a Facebook embedded, game-based PNF intervention in a three-arm, randomized controlled trial. This design allows the investigators to decompose the effects of two elements of the proposed PNF intervention; the design contrasts an alcohol use reduction only condition, and a combined alcohol use reduction with a lesbian stigma-coping condition, in contrast to a control condition. The investigators then propose a priori hypotheses derived from theory based predictions regarding different outcomes for each condition. Previous work has identified broad Facebook use in this lesbian identified population, and produced excellent retention rates with college students in PNF intervention research. Additionally, core components of the PNF alcohol intervention will be delivered to the proposed sample of lesbian identified women using what is described as an inviting, culturally-tailored, Facebook-connected social game. Finally, and very significantly, the research team has partnered with a strong and highly credible local lesbian community advocacy group. A number of refinements to the PNF intervention seeks to enhance motivation and to reduce heavy drinker defensiveness in response to the group normative drinking feedback through (1) an implementation of sharing photos from accessing participants Facebook profiles, (2) a point-based reward system, and (3) deception that the alcohol elements intervention were inserted out of a process of random selection and chance from a number of possible alternatives, rather than being of central focus of the purpose of this intervention. Many issues

diminish enthusiasm for this proposal. Previous PNF online intervention with have shown only modest intervention effect sizes, and these result shave been produced with college students, which are a very different population to the current one. Though preliminary data on the proposed refined intervention is encouraging, it is unclear if the added elements of a system of points, element of ‘chance,’ and a Facebook enabled ‘co-presence’ through use of a shared photo collage of all participants, will enhance plausibility and engagement, and further, reduce defensiveness so that it in turn increase alcohol use prevention intervention impact. Finally, in its proposed analytic approach, though the research team now consider HLM approaches (in the event of significant attrition), intent still appears to use older analysis of variance longitudinal analytic procedures such as repeated measures ANOVA; the investigator team appears to currently have limited experience with HLM/mixed regression approaches and how they can enhance precision and flexibility, even in the case of limited attrition.

1. Significance:

Strengths

- Lesbian-identified women are one of the heaviest drinking U.S. female populations, yet alcohol intervention development efforts specifically focused on lesbian communities are rare. The proposed intervention addresses a significant gap.
- The study team collected pilot data with 900 lesbian-identified women ages 21 to 40 years old, and found over 90% were active Facebook users, and that a majority of these users visited Facebook once or more per day, with no age differences in these findings. This suggests the local lesbian population that is the focus of this proposed study constitutes a broad Facebook user base, and that this attribute of high Facebook use could be leveraged in terms of reach.
- Previous work by this research group suggests PNF interventions exhibit low attrition, retaining upwards of 90% of participants, making this a promising intervention approach regarding retention.

Weaknesses

- Effect sizes for existing online PNF alcohol interventions with college students have been modest. This diminishes enthusiasm for the PNF intervention approach. The investigators note researchers have theorized these limited effects sizes in PNF interventions with college students may be due to three challenges: (1) defensive reactions to feedback by heavy drinkers, (2) difficulties in capturing participants’ attention, and (3) difficulties in establishing the credibility of the normative statistics presented. While the proposal describes enhancing user engagement by addressing these three challenges, and the 2 week outcomes presented through preliminary results that use these strategies to address these challenges appear promising, there is little in the way of peer-reviewed research documenting ability of these proposed strategies to effectively reduce defensiveness, capture attention, and establish credibility.
- In addition, this study population is developmentally different than the college student population in which these mixed PNF intervention findings are based. This sample includes older individuals, people with different drinking histories, individuals with a history of years of alcohol use, and conceivably, some individuals with a long-term history of heavy use and even disorder. The proposal does not address how or if the intervention will address the different needs of these population differences.

2. Investigator(s):

Strengths

- The PI and team have done extensive work in the area of PNF with college students, and are at the forefront of innovations intended to improve effects of PNF.

- The research team has developed relationships with a local lesbian community non-profit organization.
- Several of the investigators have significant research experience with this population, as well as in research on health and well-being among diverse sexual minority populations.
- The project has engaged consultants with strong clinical experience with this population.

Weaknesses

- Investigator, project team, or consultant depth of experience with mixed regression / HLM approaches in longitudinal analyses is not described.

3. Innovation:

Strengths

- Assessment of outcomes within the context of continued gameplay seeks to mitigate demand characteristics associated with traditional procedures of alcohol-focused follow-up surveys.
- Core components of a PNF alcohol intervention are delivered within an inviting, culturally-tailored, Facebook-connected social game. The game seeks to enhance motivation and reduce heavy drinker defensiveness associated with accurate feedback through Facebook profile photos, a point-based reward system, and elements of chance, that is, the segment of the interaction associated with drinking is presented as if it is selected by chance.
- Intervention is novel in its efforts to address and correct overestimated norms for drinking to cope with stigma associated with sexual minority status.

Weaknesses

- The integration of Facebook profile photographs and any limitations to the access of other profile information by the researcher group is not well described in the proposal. It is unclear in the proposal how or even if the Facebook connected social game is limited to access of photographs only. Because this area is not well described any concerns about how potential privacy concerns that might surface through profile embedding in the intervention are not addressed.
- If these features of intervention succeed to enhance social connectedness and credibility, it is unclear/unproven if this innovation in enhancement of these social connectedness and credibility variables will in turn increase intervention impact.

4. Approach:

Strengths

- Design decomposes the effects of two intervention elements by contrasting an alcohol only condition, and a combined alcohol and stigma-coping condition, with a control condition. Further the design proposes an a priori, theory based hypothesized set of predictions regarding the outcomes for each condition.
- A well-defined, theoretically derived set of moderator and mediator variables is proposed to be tested.

Weaknesses

- Repeated measures ANOVA is a somewhat dated analytic procedure in the contemporary peer reviewed intervention literature; given limitations in its flexibility and precision in contrast to

mixed regression/HLM procedures. Yet HLM procedures are proposed in the approach only “If attrition is a problem.”

- While it is not entirely clear as written in the presentation of this proposal, it appears that the power estimates presented for the HLM analyses seem relevant to main effects on alcohol outcomes only, but not mediation or moderator testing within an HLM framework.
- Any outcomes differences by ethnicity are unexamined in the proposed design; this is despite robust findings regarding cross-ethnic differences in alcohol attitudes and use–the design assumes no intersection of ethnic with lesbian status. These potential confounds are not modeled or studied. Clearly many the potential subgroup comparisons by ethnicity would be underpowered, however, even descriptive exploration or very basic tests of ethnic differences are not proposed.
- Basic elements of the approach for the proposed moderated-mediation modeling are not described beyond labeling them as exploratory analyses.
- Two issues surface that overlap with human subjects concerning the approach. First, the optional photo sharing appears to release personal identifying information: “participants are asked to log-in with Facebook and grant GANDR permission to use their Facebook photo. “Technically, this does not seem possible without giving profile access to the entire profile, and because of this, protections are important and not described in the proposal application. Otherwise, the project appears to ask participants to allow researchers who are strangers to allow access to their Facebook profile, and trust the project team to share photos only but no other information, and then this identifying photo will be shared with others that the person does not know, that will identify them as a sexual minority.
- The proposal describes “giving alcohol-related PNF (personal narrative feedback) the appearance of being selected by chance” when it is in fact the central intention of the intervention. Though described as elements of chance, this in fact involves deception of the research participant. While there are circumstances where deception is permitted in research, it typically involves debriefing of the participant, and at a minimum, discussion of the rational why the study is not possible without this. These are lacking from the proposal.

5. Environment:

Strengths

- Strong background in PNF intervention development.
- Significant previous work in PNF intervention studies.
- Outreach and inclusion of a nonprofit advocacy organization with credibility in the lesbian community is an integral part of the research team.

Weaknesses

- None

Protections for Human Subjects:

Unacceptable Risks and/or Inadequate Protections

- It is unclear if the informed consent will explain to participants the risk of being identified as a research participant in a study of sexual minorities through the optional photo sharing enabled by profile sharing with the researchers. There was no discussion in the informed consent procedures addressing this issue. Though the researchers appear to have taken measures to safeguard these data in their own handling and storage, this identifying information will be

shared on leaderboards and through a collage with other research participants in the study, and with no apparent way to control other participants' dissemination of this potentially identifying information regarding research participants outside of the study. True informed consent would seem to require discussion of the possibility that these photos and any other information made accessible from the Facebook profiles could be shared outside the game environment by other participants, out of the control of the researchers. Similarly, the procedures appear to involve deception. The introduction of alcohol material is presented "giving alcohol-related PNF (personal narrative feedback) the appearance of being selected by chance" when it is in fact the central purpose of the intervention and the intervention research. There are circumstances where deception is permitted in research; typically, this also involves debriefing of the participant, and at a minimum, discussion of the rational why the study is not possible without deception. These discussions are lacking from this proposal.

Data and Safety Monitoring Plan (Applicable for Clinical Trials Only):

Unacceptable

- No DSMB is described, despite several potential concerns with human subjects' issues in the procedures. The authors describe this study as a randomized controlled trial testing the efficacy of an intervention. The authors then define this study is not a Phase III clinical trial, but without justification on how this study is not.

Inclusion of Women, Minorities and Children:

- Sex/Gender: Distribution justified scientifically
- Race/Ethnicity: Distribution justified scientifically
- For NIH-Defined Phase III trials, Plans for valid design and analysis: Not applicable
- Inclusion/Exclusion of Children under 18: Excluding ages <18; justified scientifically

Vertebrate Animals:

Not Applicable (No Vertebrate Animals)

Biohazards:

Not Applicable (No Biohazards)

Applications from Foreign Organizations:

Not Applicable (No Foreign Organizations)

Select Agents:

Not Applicable (No Select Agents)

Resource Sharing Plans:

Acceptable

Authentication of Key Biological and/or Chemical Resources:

Not Applicable (No Relevant Resources)

Budget and Period of Support:

Recommended budget modifications or possible overlap identified:

- Project manager is described as a doctoral candidate in Applied Social Psychology and is listed at 100% effort on this grant application for 23.50 months of time. It is unclear through the materials how the project manager will devote 100% of effort to this project and simultaneously pursue graduate studies / maintain graduate student status.

THE FOLLOWING SECTIONS WERE PREPARED BY THE SCIENTIFIC REVIEW OFFICER TO SUMMARIZE THE OUTCOME OF DISCUSSIONS OF THE REVIEW COMMITTEE, OR REVIEWERS' WRITTEN CRITIQUES, ON THE FOLLOWING ISSUES:

PROTECTION OF HUMAN SUBJECTS: UNACCEPTABLE

- Data security measures are impressive, but clarification is needed regarding the use of Facebook user IDs, and the use of pictures as part of the intervention. It is unclear if the informed consent will explain to participants the risk of being identified as a research participant in a study of sexual minorities through the optional photo sharing enabled by profile sharing with the researchers.
- The procedures appear to involve deception. The introduction of alcohol material is presented "giving alcohol-related PNF (personal narrative feedback) the appearance of being selected by chance" when it is in fact the central purpose of the intervention and the intervention research.

INCLUSION OF WOMEN PLAN: ACCEPTABLE

INCLUSION OF MINORITIES PLAN: ACCEPTABLE

INCLUSION OF CHILDREN PLAN: ACCEPTABLE

COMMITTEE BUDGET RECOMMENDATIONS: The budget was recommended as requested.

Footnotes for 1 R21 AA025767-01A1; PI Name: LABRIE, JOSEPH W

NIH has modified its policy regarding the receipt of resubmissions (amended applications). See Guide Notice NOT-OD-14-074 at http://grants.nih.gov/grants/guide/notice-files/NOT-OD-14-074.html. The impact/priority score is calculated after discussion of an application by averaging the overall scores (1-9) given by all voting reviewers on the committee and multiplying by 10. The criterion scores are submitted prior to the meeting by the individual reviewers assigned to an application, and are not discussed specifically at the review meeting or calculated into the overall impact score. Some applications also receive a percentile ranking. For details on the review process, see http://grants.nih.gov/grants/peer_review_process.htm#scoring.

MEETING ROSTER

Epidemiology, Prevention and Behavior Research Review Subcommittee
National Institute on Alcohol Abuse and Alcoholism Initial Review Group
NATIONAL INSTITUTE ON ALCOHOL ABUSE AND ALCOHOLISM
AA-2
06/12/2017

Notice of NIH Policy to All Applicants: Meeting rosters are provided for information purposes only. Applicant investigators and institutional officials must not communicate directly with study section members about an application before or after the review. Failure to observe this policy will create a serious breach of integrity in the peer review process, and may lead to actions outlined in NOT-OD-14-073 at https://grants.nih.gov/grants/guide/notice-files/NOT-OD-14-073.html and NOT-OD-15-106 at https://grants.nih.gov/grants/guide/notice-files/NOT-OD-15-106.html, including removal of the application from immediate review.

CHAIRPERSON(S)
D'AMICO, ELIZABETH J., PHD
SENIOR BEHAVIORAL SCIENTIST
RAND CORPORATION
SANTA MONICA, CA 90407

MEMBERS
AGRAWAL, ARPANA, PHD
ASSOCIATE PROFESSOR
DEPARTMENT OF PSYCHIATRY
WASHINGTON UNIVERSITY IN ST. LOUIS
ST. LOUIS, MO 63110

ALLEN, JAMES R, PHD *
PROFESSOR
DEPT. BIOBEHAVIORAL HEALTH AND POPULATION SCIENCES
SCHOOL OF MEDICINE
UNIVERSITY OF MINNESOTA, DULUTH
DULUTH, MN 55812

BORSARI, BRIAN ERNEST, PHD
HEALTH BEHAVIOR CONCULTANT
CLINICIAN / RESEARCHER
SAN FRANCISCO VETERANS AFFAIRS HEALTH CARE SYSTEM
SAN FRANCISCO, CA 94121

CAPALDI, DEBORAH M., PHD
SENIOR SCIENTIST
DEPARTMENT OF PSYCHOLOGY
OREGON SOCIAL LEARNING CENTER
EUGENE, OR 97405

CHASSIN, LAURIE A, PHD *
REGENTS PROFESSOR
DEPARTMENT OF PSYCHOLOGY
ARIZONA STATE UNIVERSITY
TEMPE, AZ 85287

FELL, JAMES C.
PRINCIPAL RESEARCH SCIENTIST
DEPARTMENT OF ECONOMICS, JUSTICE, AND SOCIETY
NATIONAL OPINION RESEARCH CENTER AT THE UNIVERSITY OF CHICAGO
BETHESDA, MD 20814

FURR-HOLDEN, C. DEBRA, PHD
C.S. MOTT ENDOWED PROFESSOR OF PUBLIC HEALTH
PROFESSOR
DEPARTMENT OF EPIDEMIOLOGY AND BIOSTATISTICS
COLLEGE OF HUMAN MEDICINE
MICHIGAN STATE UNIVERSITY
FLINT, MI 48502

GIZER, IAN ROBERT, PHD *
ASSOCIATE PROFESSOR
DEPARTMENT OF PSYCHOLOGICAL SCIENCES
UNIVERSITY OF MISSOURI, COLUMBIA
COLUMBIA, MO 65211

MCGUE, MATTHEW K., PHD
PROFESSOR
DEPARTMENT OF PSYCHOLOGY
MEMBER, INSTITUTE OF HUMAN GENETICS
UNIVERSITY OF MINNESOTA
MINNEAPOLIS, MN 55455

NEIGHBORS, CLAYTON, PHD
PROFESSOR AND DIRECTOR
DEPARTMENT OF PSYCHOLOGY
UNIVERSITY OF HOUSTON
HOUSTON, TX 77204

READ, JENNIFER P., PHD
PROFESSOR
DEPARTMENT OF PSYCHOLOGY
UNIVERSITY AT BUFFALO
THE STATE UNIVERSITY OF NEW YORK
BUFFALO, NY 14260

TONIGAN, J SCOTT, PHD *
PROFESSOR
DEPARTMENT OF PSYCHOLOGY
UNIVERSITY OF NEW MEXICO
ALBUQUERQUE, NM 87106

XUAN, ZIMING, SCD *
ASSOCIATE PROFESSOR
DEPARTMENT OF COMMUNITY HEALTH SCIENCES
BOSTON UNIVERSITY SCHOOL OF PUBLIC HEALTH
BOSTON, MA 02118

ZEMORE, SARAH E., PHD
SENIOR SCIENTIST
CENTER ASSOCIATE DIRECTOR
DIRECTOR OF TRAINING
ALCOHOL RESEARCH GROUP
EMERYVILLE, CA 94608

SCIENTIFIC REVIEW OFFICER

GHAMBARYAN, ANNA, MD, PHD
SCIENTIFIC REVIEW OFFICER
EXTRAMURAL PROJECT REVIEW BRANCH
OFFICE OF EXTRAMURAL ACTIVITIES
NATIONAL INSTITUTE ON ALCOHOL ABUSE AND ALCOHOLISM
NATIONAL INSTITUTES OF HEALTH
ROCKVILLE, MD 20852

* Temporary Member. For grant applications, temporary members may participate in the entire meeting or may review only selected applications as needed.

Consultants are required to absent themselves from the room during the review of any application if their presence would constitute or appear to constitute a conflict of interest.
